# Supplementary material for: Performance assessment of an electrostatic filter-diverter stent cerebrovascular protection device. Is it possible not to use anticoagulants in atrial fibrilation elderly patients?
Source: Front Cardiovasc Med. 2023 Nov 29;10:1233712. doi: 10.3389/fcvm.2023.1233712 (PMC10716710; doi:10.3389/fcvm.2023.1233712)
Supplement: Supplementary file 1 [file Presentation1.pdf]

1 Appendix

1.1 Physical model

1.1.1 Blood flow model: computational fluid dynamics

Incompressible flow Blood was modeled as a Newtonian incompressible viscous fluid. The Newtonian approximation is an acceptable assumption and not far from reality in a significant part of the circulatory system under normal conditions. This is especially true in large blood vessels where red blood cells are well below the characteristic sizes of the vessels [1]. The velocity and pressure fields in the aortic arch, $\boldsymbol{u}$ and p respectively, have thus been obtained by solving the incompressible Navier-Stokes momentum and continuity equations:

$$\partial \boldsymbol{u} + \boldsymbol{u} \cdot \boldsymbol{\nabla} \boldsymbol{u} = -\frac{1}{\rho} \boldsymbol{\nabla} p + \nu \Delta \boldsymbol{u} \quad (1)$$

$$\boldsymbol{\nabla} \cdot \boldsymbol{u} = 0 \quad (2)$$

where $\rho = 1.106 \frac{g}{cm^3}$ and $\nu = 0.035 \frac{g}{cms}$ are the blood density and kinematic viscosity respectively. The corresponding boundary conditions are specified in section *Aortic arch geometry* from the main document.

Boundary conditions for the flow: healthy and atrial fibrillation conditions In order to set up the computational simulations, physical governing equations must be complemented by boundary conditions for both the fluid and particle dynamics as well as the electric field. For the fluid, the following boundary conditions are prescribed at the boundaries:

- Inlet: velocity Dirichlet boundary condition.
- Outlets: reduced-order (3-element Windkessel) model, imposed as a traction boundary condition, in which distal resistances and capacitances are modelled for a more accurate boundary conditions. In particular, the values for the present work are a 0 Pa for imposed pressure in the outlets, a resistive term equal to 100.0 and a scaling coefficient of 0.5.
- Arterial walls and deflector: no-slip Dirichlet boundary conditions.

It is noted that each one of the Windkessel boundary conditions were calibrated for the flow rates and pressures to fit the experimental results from [2] for the healthy patient case. The flowrate waveforms imposed at the inlet are representative of a healthy patient and an AF patient. In Figure 1 these two inflows used in the first stage are shown. The second stage modifies the AF inflow to adjust the physiology of a real patient in a more accurate way. Figure 1a the new inflow for AF is compared with the healthy patient. The healthy patient has a heart rate of 70 bpm and a cardiac output of $4.286\,\mathrm{L\ min^{-1}}$. In contrast, the AF patient has a higher heart rate, of 150 bpm, and a lower cardiac output, of $3.429\,\mathrm{L\ min^{-1}}$, a 20% decrease with respect to the healthy patient, as reported in [3].These flowrates were used for the second phase addapted to this specific geometry.

1.1.2 Thrombus model: particle transport

Particle transport was simulated in a Lagrangian frame of reference, following each individual particle. The main assumptions of the model were:

- particles were sufficiently small and the suspension sufficiently diluted to neglect their effect on blood-flow: i.e. one way coupling;
- particles were spherical and did not interact with each other;
- particle rotation was negligible;

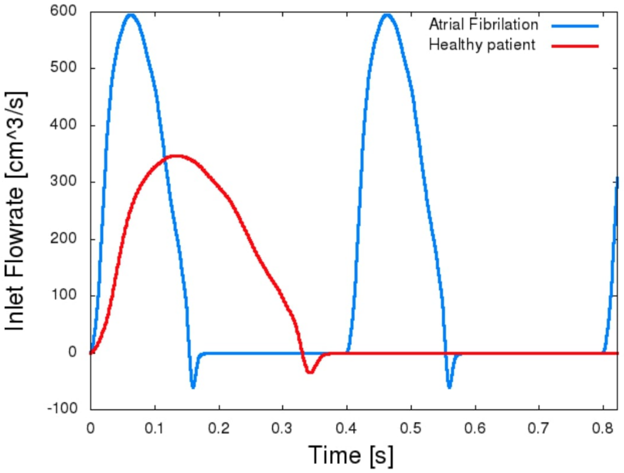

(a) Final flow rate waveforms imposed at the inlet for healthy (red) and AF (blue) patients

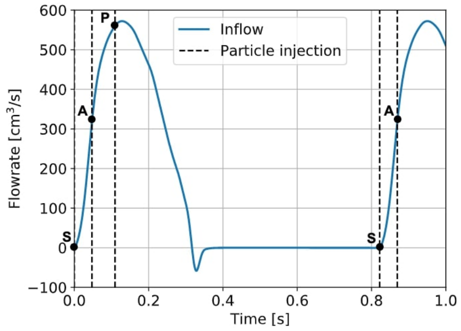

(b) Flowrate waveform showing particle injection instants in a healthy patient.

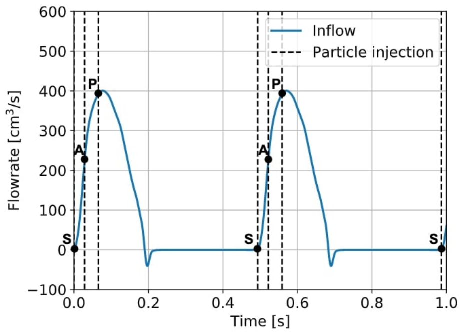

(c) Flowrate waveform showing particle injection instants in a patient with AF.

Figure 1: (a) Improved flowrate in the second stage of the work (above) and instants of particle injection, overlaid with inflow curve (below) (b) from a healthy patient and (c) from a patient with AF. Points in time S, A and P represent the beginning of systolic, accelerating, and peak stage of the cardiac cycle, respectively.

- thermophoretic forces were negligible; and
- the forces considered were drag F_d , gravitational F_g, buoyancy F_b, and, for the second stage, electric force F_e is also considered.

Particles are injected assuming a circumferential distribution at a radial distance of 40% of the aortic radius [4], as observed in Figure 2. Clot injections are conducted at three different cardiac cycle moments assumed which correspond to a beginning of systolic (S), accelerating (A), and peak stage of the cardiac cycle (P) as in [4], illustrated in Figure 1 .

In order to properly define the deflection/blocking capacity of the device, different particle types, corresponding to different particle models defined by forces considered and the diameter and density properties for each type. These different particles were used as shown in Table 1. In particular all 7 were used in the first stage and only the first 5 (from 1st to 5th of the Table 1) in the second one. Force models are applied to the particles with types 1 to 6, which have a density of $1.08\,\mathrm{g\,cm^{-3}}$ and different diameters, ranging from $d_p 10\,\mu\mathrm{m}$ to 2mm. Particles of types 1 to 3 ($d_p \in [10, 100]\mu\mathrm{m}$) are of importance for evaluating the efficacy of the device in avoiding micro-embolisms and associated SBI. Larger particles of types 4 to 6 ($d_p \in [0.5, 2]\mathrm{mm}$) represent larger embolisms, which may produce cerebral strokes.

The trajectory of these particles can be obtained by using Newton's Second Law, $\boldsymbol{F} = m\boldsymbol{a}$, where the force $\boldsymbol{F}$ is the sum of the different forces exerted on the particle, m is the particle mass

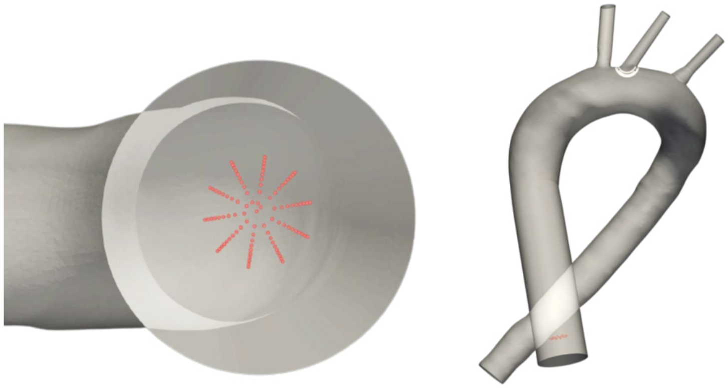

Figure 2: Zoomed view of the circumferential distribution (left) and vertical position at the inlet (right).

and $\boldsymbol{a}$ is the particle acceleration. In this case the forces applied on the particles are:

- **Gravity:** $F_g = m_p g$, where m_p and g corresponds to particle mass and gravitational acceleration.
- **Buoyancy:** $F_b = m_p g \frac{\rho_f}{\rho_p}$, where ρ_f and ρ_p corresponds to the fluid and particle densities respectively.
- **Drag:** F_d the force acting in the opposite direction to the relative motion of an object moving with respect to a surrounding fluid. The equation for the drag force assumed the particle reached its terminal velocity and is given by:

$$F_d = -8\pi\mu_f d_p C_d Re(u_p - u_f) \tag{3}$$

  being μ the dynamic flow viscosity, d_p particle density and Re the particle Reynolds number, calculated from its relative velocity with the fluid: $Re = |u_p - u_f| d_p/\nu$ with u_p and u_f corresponding to particle and fluid velocity. C_d is the drag coefficient given by Chen model described in [5]

- **Electric force:** F_e electrically charged deflector created an electric field which exerts a repulsive force through the electrical charged blood clots and described mathematically in the next section .

Particle transport was modelled by considering the above described forces, and solving Newton's second law to obtain particle accelerations:

$$a_p = \frac{F_g + F_b + F_d + F_e}{m_p} \tag{4}$$

where a_p and m_p correspond to particle acceleration and particle mass.

For the case of type 7 particles, their trajectory is obtained by computing the velocity integral, meaning that these particles act like tracers, without considering any force acting on them and they move following the flow. All particles injected in the geometry are considered spheres with their diameters described in Table 1.

It is important to remark that *in vivo* studies are more likely to show larger clot diameters[6] than those specified in the table 1. However, those large clots were not considered in our analysis as they will simply not fit between the struts. Moreover, it is important to emphasise that as indicated in Table 1, for some deflector geometries, particles of types 4, 5 and 6 have a diameter larger than or equal to the inter-strut spacing (geometries 1, 2 & 3) and thus cannot enter the LCCA through this space.

Particle type	Diameter (d_p)	$d_p \geq L_{si}$
Type 1	10 µm	None
Type 2	50 µm	None
Type 3	100 µm	None
Type 4	500 µm	Geometry 1
Type 5	1.00 mm	Geometries 1, 2 & 3
Type 6	2.00 mm	All
Type 7	-	N/A

Table 1: Particle types with their corresponding diameters (d_p) and comparison with inter-strut spacing of deflector geometries (L_{si})

.

Boundary conditions thrombus model For the particles injected into the flow, the following boundary conditions are prescribed:

- **inlet & outlets:** absorbing boundary condition, which means particles passing through such surfaces.
- **arterial walls:** slip boundary condition, which allows tangential velocities and removes the normal one and
- **deflector:** elastic bounce boundary condition, which implies imposing normal velocity with opposite sense removing the tangential momentum.

Electrostatic field The resulting electric field is obtained by solving the potential Poisson equation:

$$\nabla \cdot (\epsilon_r \nabla V) = \frac{Q_d}{\epsilon_0} \tag{5}$$

where Q_d is the total charge distributed superficially on the struts of the devices present in each configuration, ϵ_0 and ϵ_r are the electrical permittivity for the vacuum and relative permittivity of blood. Finally, we use the relation between potential and electric field: $E = -\nabla V$. The electric field generated by the superposition of the three devices deployed in the three arteries is illustrated in Figure 3.

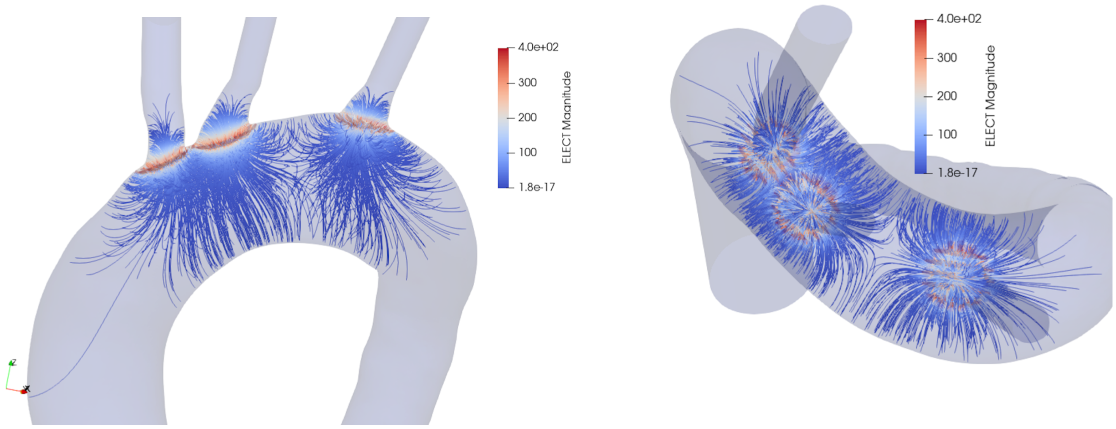

(a) Side-view of streamlines of the electric field given by the superposition of all three devices.

(b) Top-view of streamlines of the electric field given by the superposition of all three devices.

Figure 3: Streamlines of electric field generated by superposition of the 3 electrical charged devices placed in the 3 arteries

Diameters [cm]	Charges [μF]
0.001	-11.0
0.005	-55.0
0.01	-111.0
0.05	-554.0
0.1	-1108.0
0.2	-2215.0

Table 2: Particle diameters and their respective estimated electrical charges.

Boundary conditions electric potential The boundary conditions of the Poisson equation are -1.3mV in the intima or internal aortic walls and free in the inlet and outlet surfaces.

1.1.3 Electrical parameters of model

Blood flow model parameters Blood viscosity, or the thickness of blood, is generally considered to be normal when it falls within a range of 3.5 to 5.5 cP. However, it is important to note that blood viscosity is not a constant value and can vary significantly depending on the specific conditions in which it is being measured. For example, the viscosity of blood can change based on the shear rate, or the speed at which the blood is flowing. At a shear rate of $0.1\,\mathrm{s}^{-1}$, the viscosity of the same blood sample may be as high as 60 cP. However, at a shear rate of $200\,\mathrm{s}^{-1}$, the viscosity would be much lower, at around 5 or 6 cP. This means that the viscosity of blood can differ in different parts of the circulatory system, such as the large arteries, veins, and microcirculation, where shear rates can range from a few s^{-1} to over $1000\,\mathrm{s}^{-1}$. Blood viscosity is influenced by several factors, including the concentration of red blood cells, the thickness of plasma, the ability of red blood cells to deform under flow, and their tendency to clump together [7], and therefore we considered blood visocisity value of $0.035\frac{g}{cs}$

Thrombus model parameters The charge of the simulated blood clots was determined following [8], where the authors study electrophoretic mobility of electrically charged particles. Electrophoresis involves the separation of charged clots under the influence of an electric field. In fact, this mechanism is the result of the combination of two different processes, intrinsic electrophoretic mobility and electroosmotic flow. The first one can be expressed as:

$$\mu = \frac{q}{6\pi\nu r_s} \tag{6}$$

where μ is the electrophoretic mobility, q is the charge of the particle that we are interested in, ν the viscosity of the medium, and r_s the hydrodynamic radius. The electrophoretic mobility was taken as $\mu = -0.0336\,\mathrm{cm}^2\,\mathrm{statV}^{-1}\mathrm{s}^{-1} = -1.12\,\mathrm{\mu m\,s\,V}^{-1}\,\mathrm{cm}^{-1}$[9]. The densities assigned to blood clots were $1.08\,\mathrm{g\,cm}^{-3}$ [10]. By expressing the charge as a function of the remaining parameters in (6), electrical charges were obtained for each diameter, and summarized in Table 2.

Device model parameters As described in Section **??**, the surface charge on the graphene oxide in $0.001MNaCl$ was determined approximately as $-75\,\mathrm{mC\,m}^{-2}$ in good agreement with the previous work [11] where the value was equal $-80\,\mathrm{mC\,m}^{-2}$, at pH 6.5 extracted from a zeta potential measurement, albeit in NaCl. In CGS units, these two values correspond to $-22,484.43\,\mathrm{statC\,cm}^{-2}$ and $-23,983.39\,\mathrm{statC\,cm}^{-2}$. Based on these results, we have considered a surface charge of the device corresponding to $-24,000\,\mathrm{statC\,cm}^{-2}$. This value needs to be integrated for all the surfaces of the devices placed in the domain producing the total charge Q_d in (5), responsible for the electric field, which exerts the repulsive force on blood clots.

1.2 Quantities of interest

The following quantities of interest are used for both particle statistics and flow dynamics:

- The particle deflecting efficacy at the arterial outlets where the device is deployed, with and without considering electric charge. This metric provides a means of computing the efficacy of the device CEPD.
- The maximum ejected flowrate in the treated artery. This value can be used to determine the variation in flowrate ejected to the arteries with respect to the case without deflector. It thus enables computing what will be the effect on oxygen delivery to the cerebrovascular system.
- Volume-averaged metrics have also been computed in order to evaluate the risk of thrombus formation due to the presence of the medical device. The region of interest considered is the volume encapsulated by the deflector as shown in Figure 4. The quantities of interest evaluated are:
  - The average kinetic energy, associated to washout.
  - The average second invariant of the velocity gradient $\boldsymbol{\nabla u}$, that is, $Q = \frac{1}{2}\left(||\boldsymbol{\Omega}||^2 - ||\boldsymbol{S}||^2\right)$, where $\boldsymbol{S}$ is the symmetric part of the velocity gradient known as the rate of strain, and $\boldsymbol{\Omega}$ is the antisymmetric part known as the vorticity tensor. This invariant, is also used in the Q-criterion that quantifies the level of vorticity in the flow by defining vortices as volumes where $Q > 0$ (where the vorticity magnitude is greater than the magnitude of the rate of strain).
  - ΔQ:the drop in ejected flowrate at the arterial outlets where the device is deployed, relative to the untreated case.

1.3 Hydrodynamic quantities of interest

The first stage of the present work is focused on the analysis of the hydrodynamics effects of the deflector placed at the base of the arteries and how these hydrodynamics effects affect the trajectories of the potential clots travelling through the aortic arch. To that end, different configurations of the struts forming the presented device are analysed.

The presence of the deflector influences the haemodynamic flow locally. Figure 5 qualitatively depicts the fluid velocity field around the LCCA base where the deflector is deployed in this first study. Different instants of a single cycle are shown corresponding approximately to the peak stage (Figure 5a), the midterm of the deceleration phase (Figure 5b) and the plateau stage (Figure 5c). To quantitatively analyse the effect of the deflector on the flow, different hydrodynamic metrics were computed.

Considering the results of the second of the cited metrics in the supplementary appendix section 1.2, the maximun flowrate, we could observe that the mean value all the configurations is 68,91 with a standard deviation of 1.019. So no significant reduction in flowrate is obtained in either case for the different configurations represented in the table presented in section *Device design* from the main document.

The kinetic energy quantity of interest evaluated in the region of interest considered in Figure 4 is reduced comparing the non-device configuration for all the analyzed configuration, obtaining a maximum value of 20% for the Geometry-4 case represented in the table presented in section *Device design* from the main document. Although the results indicates that the presence of the deflector reduces the velocity downstream of the deflector, the Geometry-2 configuration is the one that mantain higher kinetic energy with a reduction of less that 10 % .

In order to quantify the degree of vortices generated by the deflector, the Q-invariant (second invariant of velocity gradient) is computed. Based on the results obtained, again we see that the deflector presenting a lowest Q value (*i.e.*, presenting similar results to the case with no deflector

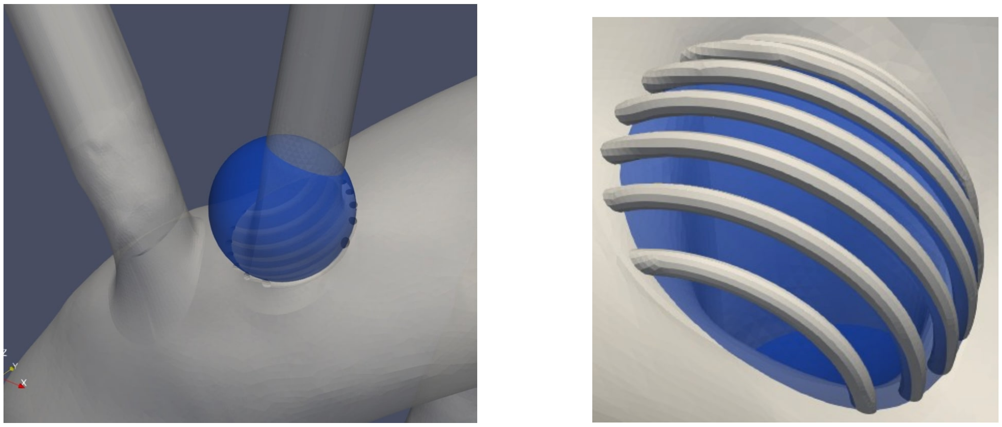

(a) (b)

Figure 4: Region of interest where volumetric quantities of interest are integrated.

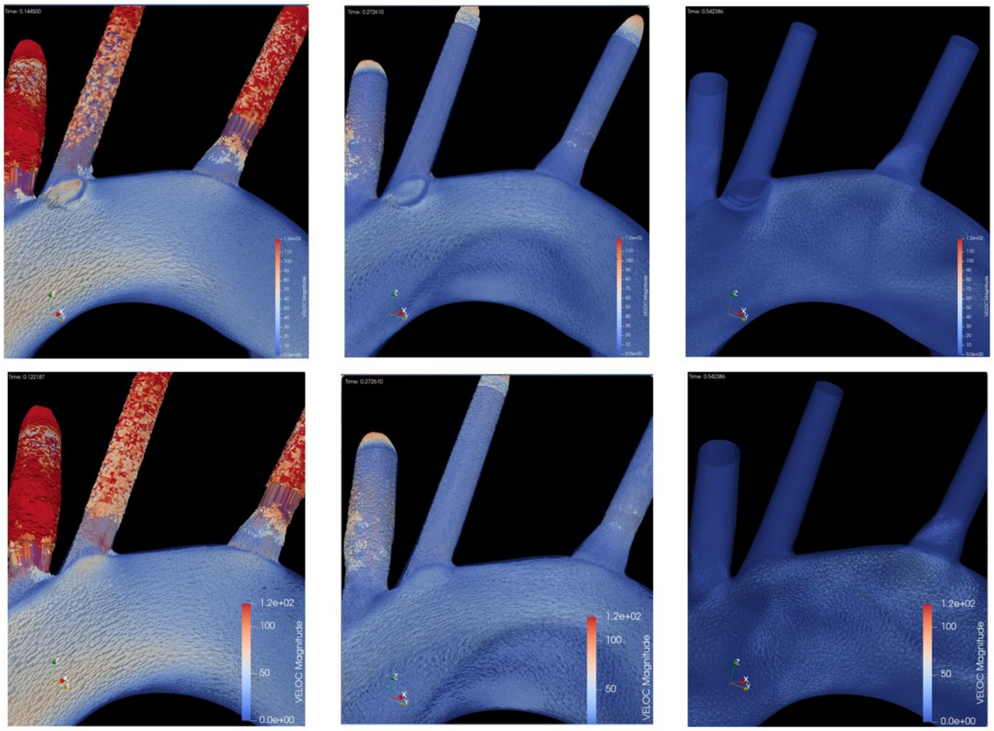

Figure 5: Velocity field around LCCA artery with deployed deflector (top) and without deflector device (bottom) for different instants of a single cycle, from left to right: (a) $t = 0.1$s, (b) $t = 0.27$s, and (c) $t = 0.54$s.

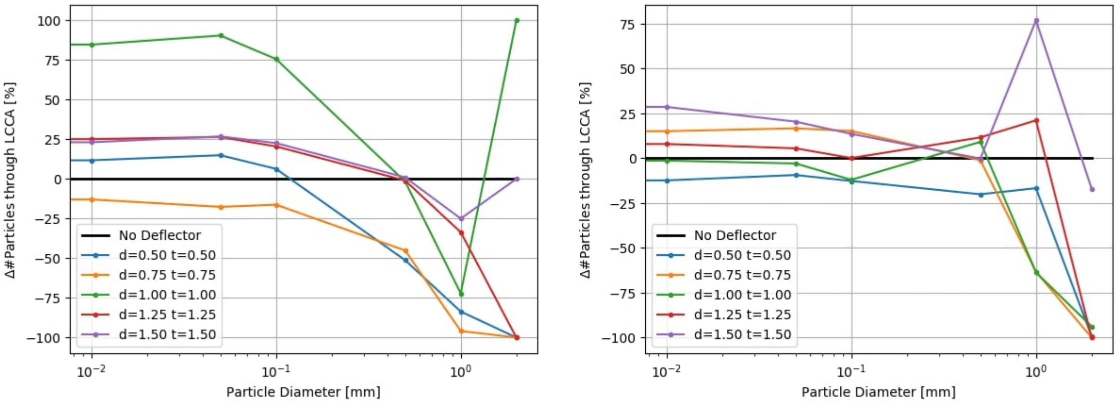

(a) Healthy.

(b) Atrial fibrillation.

Figure 6: Variation in number of particles entering the LCCA for increasing strut thicknesses for the (a) healthy, and AF patient. Particle depositions are normalised by the no-deflector case $L_{si} = 0$mm. Inter-strut spacings are equal to the strut thickness in each case, as can be seen in the table of section *Device design* from the main document.

implanted) is the one with dimensions $L_{si} = 0.75\,\mathrm{mm}$ and $L_{st} = 0.75\,\mathrm{mm}$, being the design which produces less vortices in the evaluated region.

1.4 Results for the first stage

1.4.1 Healthy Patient: Comparison between all different morphologies over LCCA

Figure 6 shows results for the particles effectively passing through the deflector device (in between struts) deployed at the base of the LCCA. Particle counts are normalised by the total number of particles reaching the LCCA outflow in the no-deflector case, obtaining our quantity of interest called ϵ. It can be seen that particle counts there are only reduced for particles above or equal to 0.5 mm for the healthy patient case (Figure 6a). These sizes correspond to the minimum inter-strut spacings of the analysed designs. As should occur, particles above the inter-strut spacing are 100% blocked, while when slightly below the inter-strut spacing (*e.g.*, see Figure 6a: $d_p 0.5\,\mathrm{mm}$, $L_{si} = 0.75\,\mathrm{mm}$ where particle deposition is reduced by 38%), a moderate reduction is observed. For particles well below the inter-strut spacing ($d_p \ll L_{st}$) no significant reduction is observed. In fact, for some of these particle-strut combinations an increased number of particles are detected in the LCCA. It is remarkable that reductions in particles passing through the device are observed for particles with diameters below L_{si} in the $L_{si} = 0.75\,\mathrm{mm}$ in the healthy case (Figure 6a).

1.4.2 Atrial Fibrilation patient: Comparison between all different morphologies over LCCA

In this case, as we can see in Figure 6b, particle counts there are only reduced for particles above or equal to 1.0mm for the AF case. The reductions in particles passing through the device are observed in Figure 6b with diameters below $L_{si} = 0.5$mm in this AF case.

Device position	Particle diameter	BCT		LCCA		LSA	
		Neutral	Charged	Neutral	Charged	Neutral	Charged
Baseline	10 µm	29,508	N/A	30,452	N/A	36,010	N/A
Baseline	50 µm	29,788	N/A	30,781	N/A	36,574	N/A
Baseline	100 µm	30,720	N/A	31,843	N/A	38,339	N/A
Baseline	500 µm	56,328	N/A	49,134	N/A	44,508	N/A
Baseline	1 mm	103,285	N/A	34,010	N/A	10,713	N/A
BCT	10 µm	29,171	7,880	31,088	34,799	33,499	35,432
BCT	50 µm	29,452	12,956	31,480	35,595	33,985	35,578
BCT	100 µm	30,232	17,983	32,651	36,226	35,458	37,149
BCT	500 µm	46,287	39,422	58,550	58,325	45,096	52,041
BCT	1 mm	0	0	134,441	132,285	13,494	15,385
LCCA	10 µm	31,354	32,618	29,101	1,384	30,071	29,852
LCCA	50 µm	31,646	33,044	29,421	6,250	30,552	31,475
LCCA	100 µm	32,619	36,278	30,288	12,346	32,019	33,408
LCCA	500 µm	58,608	59,157	39,431	31,330	51,892	58,812
LCCA	1 mm	104,238	104,238	0	0	41,472	43,227
LSA	10 µm	34,599	34,596	35,818	35,890	31,806	2,702
LSA	50 µm	34,923	34,921	36,365	36,427	32,268	8,601
LSA	100 µm	35,967	35,969	37,527	37,554	34,008	19,172
LSA	500 µm	65,852	65,859	57,769	57,798	44,754	42,742
LSA	1 mm	116,742	116,697	38,899	38,834	0	0
All	10 µm	29,772	8,931	29,008	1,694	28,388	2,622
All	50 µm	30,069	14,043	29,372	7,462	28,811	7,542
All	100 µm	30,861	19,476	30,330	15,689	30,350	17,389
All	500 µm	46,723	40,154	49,470	40,851	50,963	50,220
All	1 mm	0	0	0	0	0	0

(a) Particle counts for the healthy patient deploying the device in single arteries (BCT, LCCA, and LSA), and in all at once.

1.4.3 Deflector deployed in a single artery

Analogously to previous section, the deflector was deployed individually in each aortic arch artery (BCT, LCCA, or LSA), running separate simulations for each case (see figure representing different device deployment configuration from section *Device design* from the main document), prescribing the AF flow rate at the inlet. The observations noted in the healthy patient are still observed for the AF patient, with similar particle count differentials when implanting the deflector, and when considering electrical charge:

1. the neutral particles are filtered with an efficacy of 32.0% on average in the artery where the deflector is deployed (26.0% for the healthy patient),

2. this average efficacy increases to 59.6% when the particles are charged (66.5% for the healthy patient),
3. the deflection efficacy of the electrical charge diminishes for larger particle sizes,
4. the number of particle counts of arteries distal to the artery where the deflector is deployed increases by 37.3% on average with respect to the baseline case (38.8% for the healthy patient), and
5. they increase to 41.6% when the particles are electrically charged (46.6% for the healthy patient).

In summary, as for the healthy patient, in the AF patient, the deflector effectively reduces the particle counts in the treated arteries, while producing a slight increase of the particles entering distal arteries left open.

Device position	Particle diameter	BCT		LCCA		LSA	
		Neutral	Charged	Neutral	Charged	Neutral	Charged
Baseline	10 µm	21,975	N/A	21,375	N/A	26,335	N/A
Baseline	50 µm	22,422	N/A	21,799	N/A	26,948	N/A
Baseline	100 µm	23,448	N/A	23,086	N/A	28,605	N/A
Baseline	500 µm	48,580	N/A	42,855	N/A	43,153	N/A
Baseline	1 mm	98,866	N/A	303	N/A	1,682	N/A
BCT	10 µm	19,664	8,115	25,245	27,067	30,354	31,226
BCT	50 µm	20,047	12,043	25,794	27,808	31,000	31,809
BCT	100 µm	20,888	15,024	27,436	29,486	32,701	33,514
BCT	500 µm	40,872	38,302	53,302	53,179	37,337	40,050
BCT	1 mm	0	0	124,319	123,935	14,264	15,737
LCCA	10 µm	24,143	25,018	20,525	5,023	21,352	21,922
LCCA	50 µm	24,689	27,002	20,749	9,742	21,916	22,851
LCCA	100 µm	25,970	29,661	21,242	12,657	23,770	24,549
LCCA	500 µm	49,661	50,317	24,865	22,992	51,114	52,066
LCCA	1 mm	94,257	94,815	1	0	44,185	44,973
LSA	10 µm	24,108	24,099	22,842	22,826	22,078	5,323
LSA	50 µm	24,663	24,658	23,178	23,153	22,352	10,686
LSA	100 µm	25,652	25,836	24,378	24,391	23,465	17,572
LSA	500 µm	53,123	53,129	38,096	38,068	33,318	31,989
LSA	1 mm	99,464	99,460	26,973	27,006	0	0
All	10 µm	23,289	10,012	22,265	5,671	23,242	5,056
All	50 µm	23,550	15,317	22,762	12,291	23,633	10,291
All	100 µm	24,650	21,099	23,579	17,115	24,980	16,841
All	500 µm	40,167	38,838	36,971	34,242	41,908	41,060
All	1 mm	0	0	0	0	0	0

(a) Particle counts for the AF patient deploying the device in single arteries (BCT, LCCA, and LSA), and in all at once.

Table 4: Particle filtering efficacies for the AF patient

References

[1] T. Sochi. Non-newtonian rheology in blood circulation. *arXiv: Fluid Dynamics*, 2013.

[2] M. Bonfanti, G. Franzetti, S. Homer-Vanniasinkam, V. Díaz-Zuccarini, and S. Balabani. A combined in vivo, in vitro, in silico approach for patient-specific haemodynamic studies of aortic dissection. *Annals of biomedical engineering*, 48:2950–2964, 09 2020.

[3] J.S. Alpert, P. Petersen, and J. Godtfredsen. Atrial fibrillation: natural history, complications, and management. *Annual review of medicine*, 39:41–52, 1988.

[4] H.W. Choi, J. Navia, and G. Kassab. Stroke propensity is increased under atrial fibrillation hemodynamics: A simulation study. *PloS one*, 8:e73485, 09 2013.

[5] N-J. Cheng. Comparison of formulas for drag coefficient and settling velocity of spherical particles. *Powder Technology*, 189(3):395–398, 2009.

[6] D. Barbut, F-S.F. Yao, Y-W. Lo, R. Silverman, D.N. Hager, R.R Trifiletti, et al. Determination of size of aortic emboli and embolic load during coronary artery bypass grafting. *The Annals of Thoracic Surgery*, 63(5):1262–1265, 1997.

[7] E. Nader, S. Skinner, M. Romana, R. Fort, N. Lemonne, N. Guillot, et al. Blood rheology: Key parameters, impact on blood flow, role in sickle cell disease and effects of exercise. *Frontiers in physiology*, 10:1329, 2019.

[8] A.M. Stalcup. Chapter 8 - chiral separations by capillary electrophoresis. In K.W. Busch and M.A. Busch, editors, *Chiral Analysis*, pages 241–275. Elsevier, Amsterdam, 2006.

[9] K.M. Jan and S. Chien. Role of surface electric charge in red blood cell interactions. *J Gen Physiol.*, 61(5):638–54, 1973.

[10] V.M. Nahirnyak, S.W. Yoon, and C.K. Holland. Acousto-mechanical and thermal properties of clotted blood. *The Journal of the Acoustical Society of America*, 119(6):3766–3772, 2006.

[11] W.J. Paschoalino, N.A. Payne, T.M. Pessanha, S.M. Gateman, L.T. Kubota, and J. Mauzeroll. Charge storage in graphene oxide: Impact of the cation on ion permeability and interfacial capacitance. *Analytical chemistry*, 92(15):10300–10307, 2020.
