# Supplementary figures and images for: Performance assessment of an electrostatic filter-diverter stent cerebrovascular protection device. Is it possible not to use anticoagulants in atrial fibrilation elderly patients?
Source: Front Cardiovasc Med. 2023 Nov 29;10:1233712. doi: 10.3389/fcvm.2023.1233712 (PMC10716710; doi:10.3389/fcvm.2023.1233712)

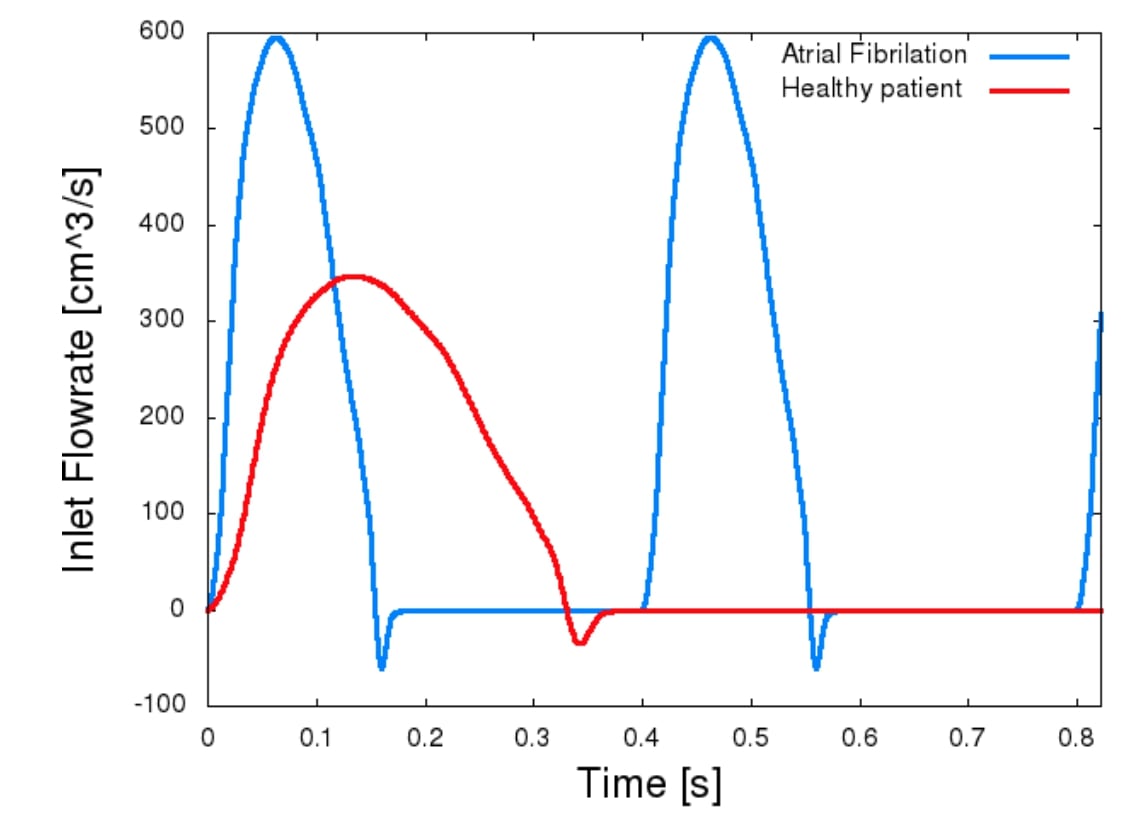

Supplement: Supplementary file 2 [file Image1.jpeg]

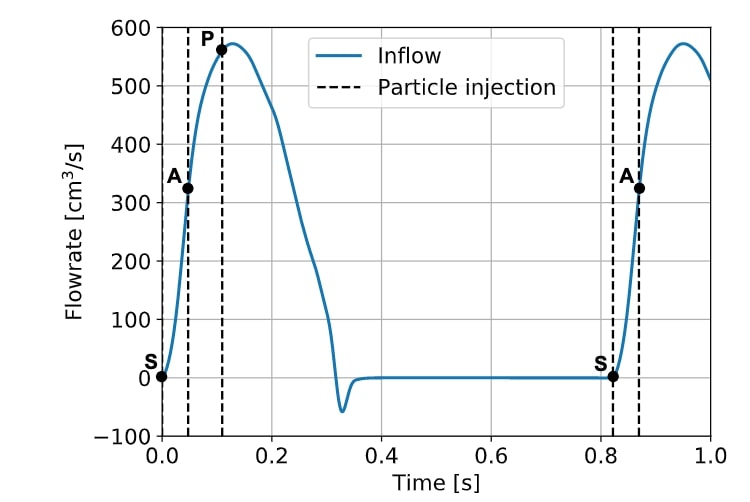

Supplement: Supplementary file 3 [file Image2.jpeg]

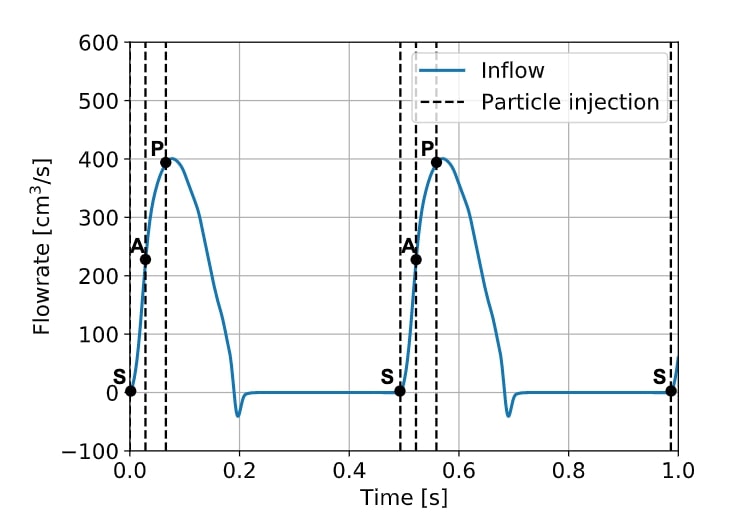

Supplement: Supplementary file 4 [file Image3.jpeg]

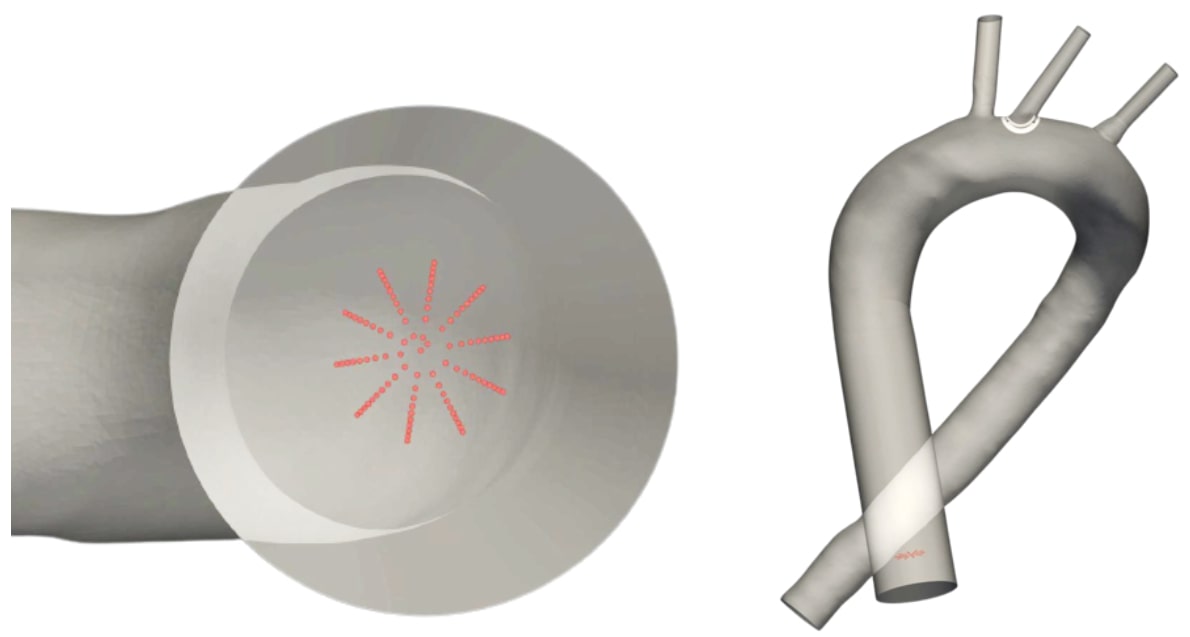

Supplement: Supplementary file 5 [file Image4.jpeg]

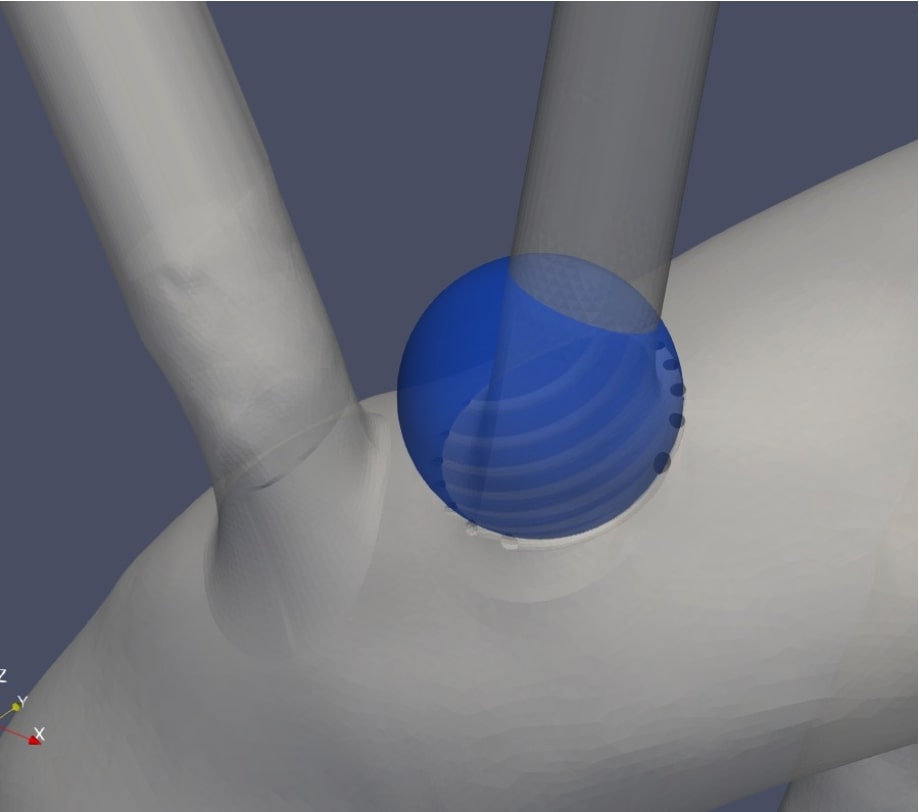

Supplement: Supplementary file 6 [file Image5.jpeg]

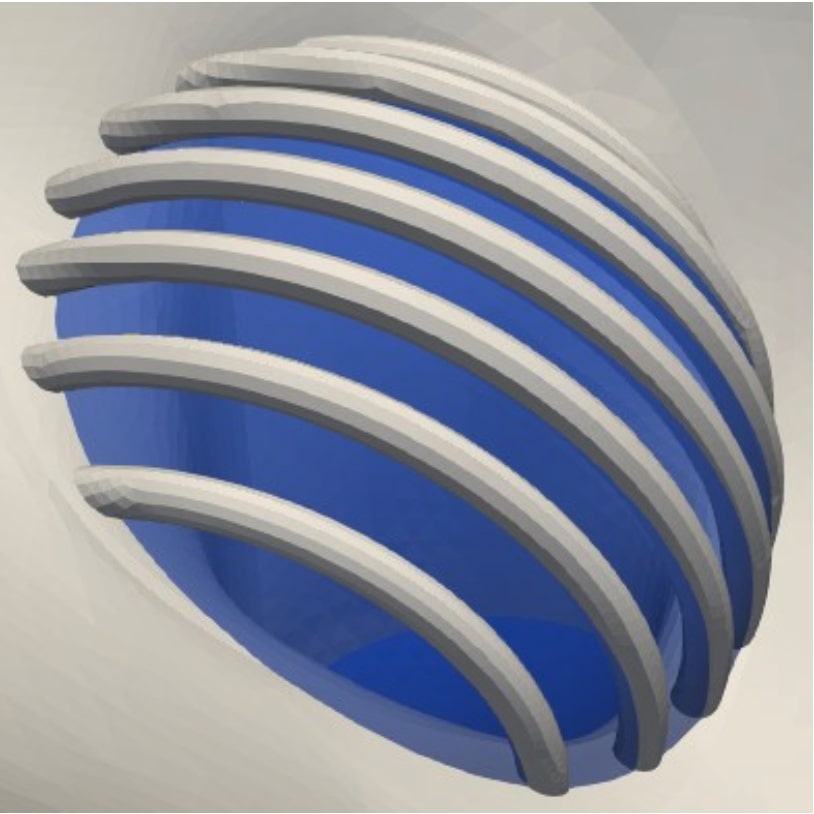

Supplement: Supplementary file 7 [file Image6.jpeg]

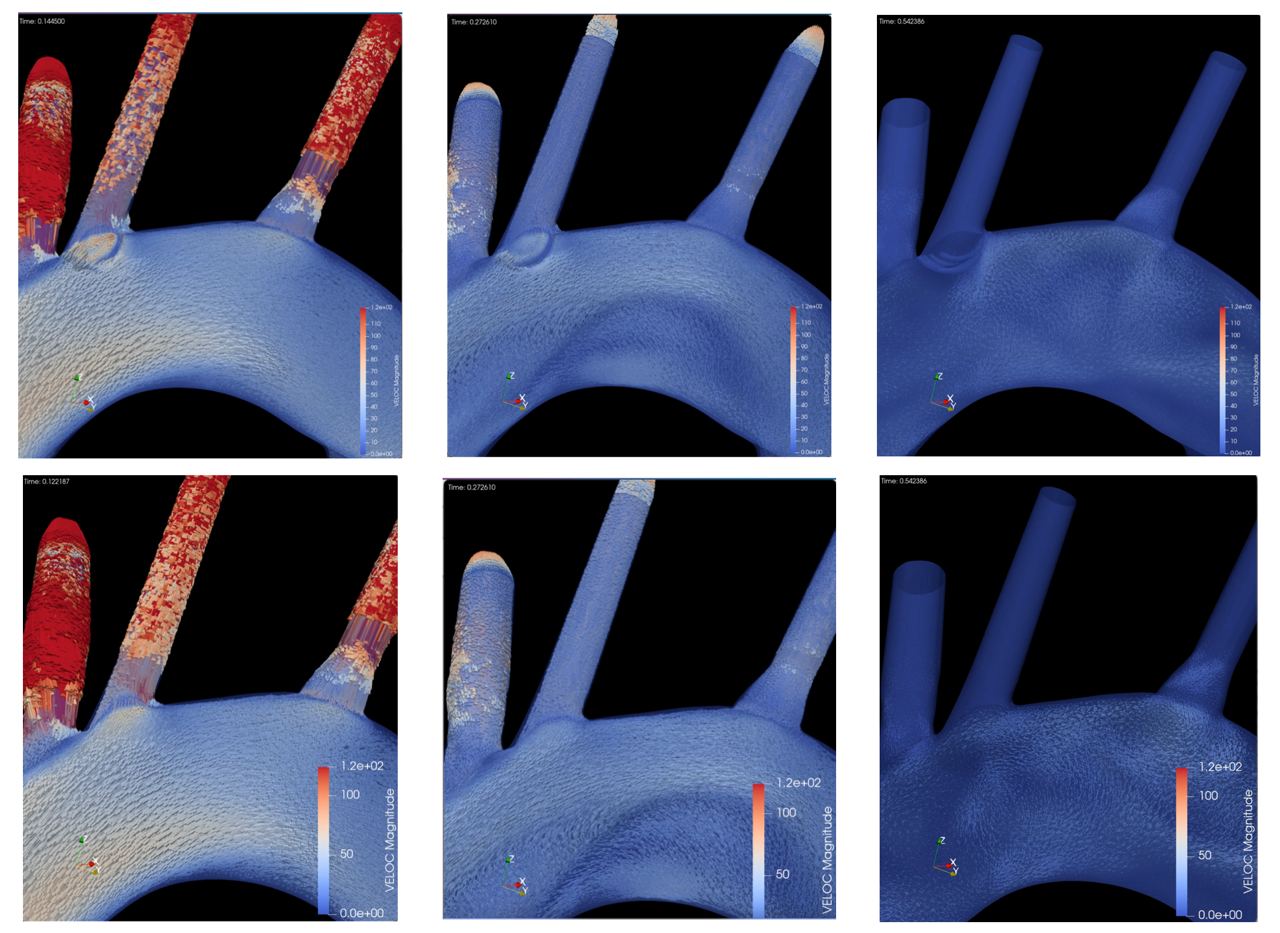

Supplement: Supplementary file 8 [file Image7.jpeg]

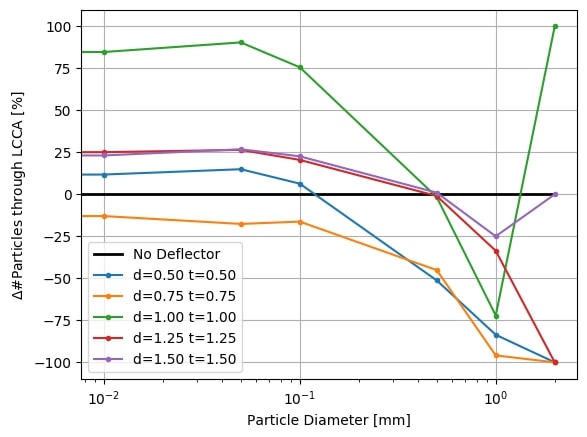

Supplement: Supplementary file 9 [file Image8.jpeg]

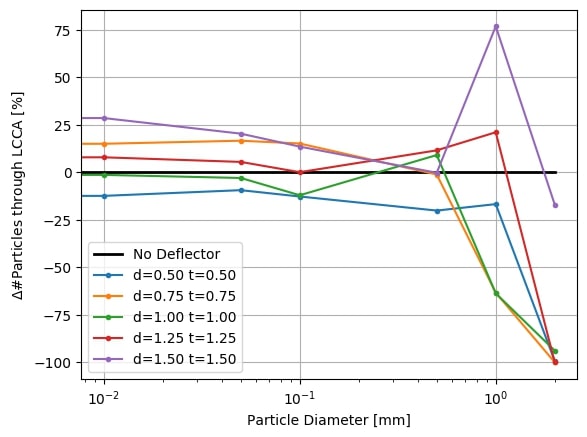

Supplement: Supplementary file 10 [file Image9.jpeg]

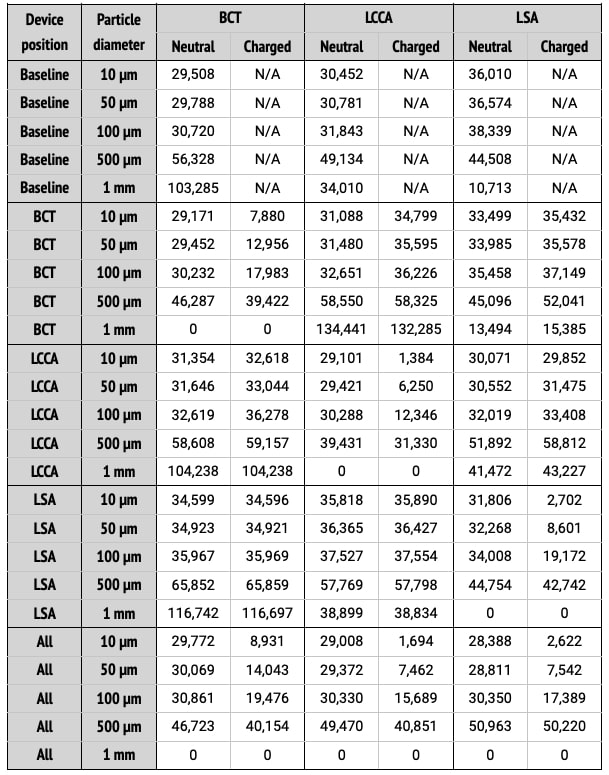

Supplement: Supplementary file 11 [file Image10.jpeg]

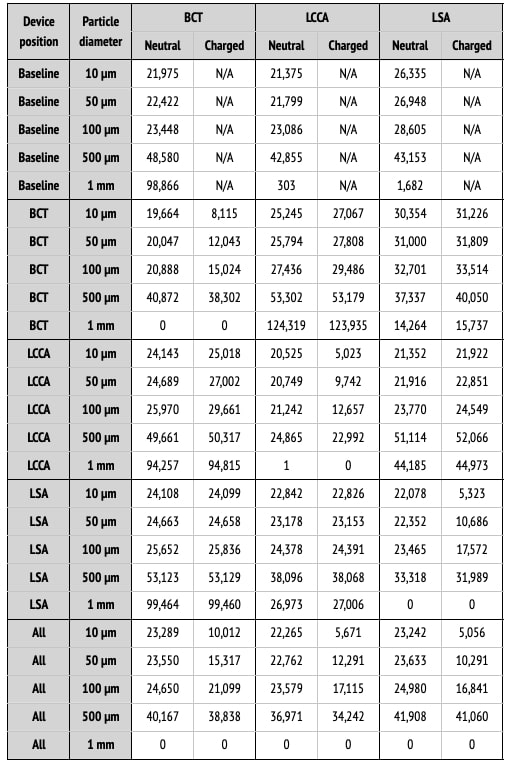

Supplement: Supplementary file 12 [file Image11.jpeg]
